# GDF15 Repression Contributes to 5-Fluorouracil Resistance in Human Colon Cancer by Regulating Epithelial-Mesenchymal Transition and Apoptosis

**DOI:** 10.1155/2020/2826010

**Published:** 2020-09-28

**Authors:** Bin Wang, Nina Ma, Xixi Zheng, Xiao Li, Xiao Ma, Jiexuan Hu, Bangwei Cao

**Affiliations:** ^1^Department of Medical Administration, Beijing Friendship Hospital, Capital Medical University, Beijing, 100050, China; ^2^Department of Oncology, Beijing Friendship Hospital, Capital Medical University, Beijing, 100050, China

## Abstract

Chemotherapy based on 5-fluorouracil (5-FU) is the standard approach for colon cancer treatment, and resistance to 5-FU is a significant obstacle in the clinical treatment of colon cancer. However, the mechanisms underlying 5-FU resistance in colon cancer cells remain largely unknown. This study aimed at determining whether 5-FU-resistant colon cancer cells undergo epithelial-mesenchymal transition (EMT) and apoptosis and the role of GDF15—a member of the transforming growth factor *β*/bone morphogenetic protein super family and a protein known to be involved in cancer progression—in the regulation of EMT and apoptosis of these cells, along with the underlying mechanisms. In vitro apoptosis detection assay, growth inhibition assay, transwell, and wound healing experiments revealed that 5-FU-resistant colon cancer cells possessed enhanced EMT and antiapoptotic ability. These cells also showed a stronger tendency to proliferate and metastasize in vivo. Quantitative reverse transcription-PCR and western blotting revealed that 5-FU-resistant colon cancer cells expressed lower levels of growth differentiation factor 15 (GDF15) than did 5-FU-sensitive colon cancer cells. Moreover, the transient *GDF15* overexpression resensitized 5-FU-resistant colon cells to 5-FU. Collectively, these findings indicate the mechanism underlying the 5-FU resistance of colon cancer cells and provide new therapeutic targets for improving the prognosis of colon cancer patients.

## 1. Introduction

Resistance to 5-fluorouracil (5-FU) is the primary reason for the failure of traditional chemotherapy for colon cancer [1]. Despite significant advances in our understanding of 5-FU resistance, the underlying molecular mechanisms are not fully characterized. Evidence indicates that epithelial-mesenchymal transition (EMT) and apoptosis are involved in tumor progression [2, 3] and that drug-resistant cells often show accelerated proliferation and distant metastasis [4, 5]. These findings suggest that both EMT and apoptosis are possibly involved in the development of chemotherapy resistance. Our previous results showed that growth differentiation factor 15 (GDF15) is downregulated in HCT-15/FU drug-resistant colon cancer cells compared with drug-sensitive colon cells; these cells also showed accelerated proliferation and angiogenesis in vitro and in vivo. These results suggested that GDF15 may play a crucial role in the regulation of 5-FU sensitivity of colon cancer cells. However, the exact mechanism remains unknown.

GDF15, a member of transforming growth factor *β* (TGF-*β*)/bone morphogenetic protein (BMP) superfamily, is expressed in many types of tissues and has gained extensive attention with the increase in research on cancer progression [6, 7]. However, the effects of GDF15 on tumor development are not well understood and are contradictory [6]. Like other TGF-*β* members, GDF15 can play a variety of biological roles via the regulation of the TGF-*β*/Smad signaling pathway. Previous studies have shown that GDF15 can promote EMT and metastasis in colorectal cancer [8] and contribute to radioresistance and cancer stemness of head and neck cancer [9]. However, the role of GDF15, especially in 5-FU resistance in colon cancer, is not yet fully understood.

In this study, we determined whether 5-FU-resistant colon cancer cells undergo EMT and apoptosis. Additionally, we investigated whether GDF15 is involved in the regulation of EMT and apoptosis and explored the underlying mechanisms. Our results show that the low expression of GDF15 contributes to 5-FU resistance in human colon cancer by regulating EMT and apoptosis via a Smad-associated signaling pathway. Thus, our findings will provide new insights into 5-FU resistance in colon cancer and shed light on a target for colon cancer gene therapy.

## 2. Materials and Methods

### 2.1. Cell Culture

HCT-15 (human colon cancer cells) and HCT-15/FU cells were bought from the cell bank of Chinese Academy of Sciences (Shanghai, China) and were cultured in RPMI 1640 medium containing 10% fetal bovine serum (FBS, Gibco, USA). HCT-15/FU cells were supplemented with 3.2 *μ*g/ml 5-FU (the initial concentration of 5-FU was determined with the half maximal inhibitory concentration (IC_50_) of HCT-15 cells, starting at 1/3 of IC_50_ and increasing by 1/6-1/3 each time; the cells were cultured for 2-4 times at each concentration until stable HCT-15/FU cell line was obtained). All cells were incubated at 37°C with 5% CO_2_.

### 2.2. GDF15 Overexpression

HCT-15/FU cells were seeded and transfected with a GDF15-overexpressing vector pCMV-GDF15 (YouBioTech, China) or the empty pCMV vector (YouBioTech, China) as control.

### 2.3. Cell Proliferation Assay

In total, 5 × 10^3^ HCT-15 or HCT-15/FU cells were cultured in each well of a 96-well plate and treated with various concentrations of 5-FU (the concentrations of 5-FU were 0, 0.1, 0.2, 0.4, 0.8, 1.6, and 3.2 mM for HCT-15 cells and 0, 1, 2, 4, 8, 16, and 32 mM for HCT-15/5-FU cells). The viability of the cells was measured at the time point of 0 and 48 h using the MTS reagent (CellTiter 96® Aqueous One Solution Cell Proliferation assay, Promega). The optical density was measured at 490 nm after being incubated at 37°C for 2 h using an enzyme-labeled meter (Spectramax M3; Molecular Devices). Using GraphPad Prism 5.0 software to calculate the inhibition rate and draw a concentration effect curve, three independent tests were conducted for the cell proliferation assay.

### 2.4. Cell Migration Assay

The migration ability of HCT-15 or HCT-15/FU cells was tested in a Transwell Boyden Chamber (8 mm pore size, 6.5 mm diameter) seeded in the upper chamber with 1 × 10^4^ cells in 0.5 ml serum-free medium with 0.76 mM 5-FU, and the lower chamber was filled with 0.8 ml medium containing 10% FBS. After incubating for 48 h, cells were fixed with 100% methanol for 20 min and stained with 0.1% crystal violet for 15 min. The cells in the upper compartment were wiped off. Images were taken using a photomicroscope (Nikon, Japan) and quantified by counting at least three fields.

### 2.5. Wound Healing Assay

HCT-15 or HCT-15/FU cells were cultured in 24-well plates. At 100% confluent, a 20 *μ*L pipette tip was used to scratch a wound at the centre of the cell monolayer in the culture plates. The wounds between cells were washed twice with PBS and cultured with culture medium containing 0.76 mM 5-FU for another 48 h. Images of wounds were captured at 0, 12, 24, 36, and 48 h, and the wound healing area was calculated by ImageJ software.

### 2.6. RNA Extraction and Quantitative Reverse Transcriptase-PCR (qRT-PCR)

Total RNA was extracted from cultured cells using the TRIzol Reagent (Invitrogen) according to the manufacturer's instructions. cDNA was obtained from RNA using a reverse transcriptase kit (Takara, China), and quantitative real-time PCR was performed with SYBR Premix ExTaqTM II (Takara, Dalian, China). The cycling conditions were 25°C for 10 min and 37°C for 2 min, followed by 85°C for 5 min. The mRNA levels were normalized to GAPDH, and 2^-*ΔΔ*Ct^ was used for data statistics [10]. The primers used in qRT-PCR are GDF15 (forward: 5′-GACCCTCAGAGTTGCACTCC-3′; reverse: 5′-GCCTGGTTAGCAGGTCCTC-3′) and GAPDH (forward: 5′-GGAGCGAGATCCCTCCAAAAT-3′; reverse: 5′-GGCTGTTGTCATACTTCTCATGG-3′).

### 2.7. Western Blotting Assay

Whole proteins were extracted from cells using immunoprecipitation buffer and quantified using the BCA protein assay kit (Thermo scientific Pierce). After separation on a 10% SDS-PAGE gel, proteins were transferred to a PVDF membrane (Millipore) and blocked in 5% nonfat milk. Proteins were incubated with primary antibodies overnight at 4°C and secondary antibodies at room temperature for 2 h. TBST buffer was used to wash off the unbound antibodies. Protein bands were visualized by an ECL plus system (Beyotime). The specific primary antibodies used in western blot analysis were as follows: anti-MDR1 (1 : 1000, 13342, CST), anti-MRP1 (1 : 1000, 72202S, CST), anti-E-cadherin (1 : 5000, Cat. No. 20874-1-AP, Proteintech), anti-N-cadherin (1 : 5000, ab76011, Abcam), anti-BAX (1 : 1000, ab32503, Abcam), anti-Bcl-2 (1 : 2000, ab182858, Abcam), anti-cleaved caspase-3 (1 : 1000, 9664S, CST), anti-MMP14 (1 : 2000, ab51074, Abcam), anti-p-Smad2/3 (1 : 500, Hangzhou HuaAn Biotechnology), anti-Smad2/3 (1 : 1000, Hangzhou HuaAn Biotechnology), and anti-GAPDH (1 : 5000, Cat. No. 60004-1-Ig, Proteintech).

### 2.8. Apoptosis Detection

Apoptosis assays were conducted according to the manufacturer's instructions (BD Biosciences, San Jose, CA, USA). The cells were analyzed by a FACS Verse (Becton-Dickinson, Franklin Lakes, NJ, USA) after incubation in the dark at 4°C for 15 min.

### 2.9. Establishment of Tumor in Nude Mice

Eight male BALB/c-nu mice (4–6 weeks old) were randomly divided into two groups. The mice were inoculated subcutaneously with HCT-15 or HCT-15/FU cells (1 × 10^7^ cells in 100 *μ*L PBS, respectively) in the groin. One week later, 0.76 mM 5-FU was injected into developed tumor every other day. After 28 days, all the mice were sacrificed by cervical dislocation, and the tumors were excised, measured, weighed, and photographed. All animal experiments were performed in accordance with a protocol approved by the experimental animal welfare ethics review committee of National Institutes for Food and Drug Control, China.

### 2.10. Statistical Analysis

Statistical analysis was conducted using GraphPad Prism 5.0 software. Data were expressed as the mean ± SEM. The differences between two groups were analyzed by Student's *t*-test, and three groups or more were analyzed by one-way ANOVA. *P* < 0.05 was considered statistically significant.

## 3. Results

### 3.1. 5-FU-Resistant Colon Cancer Cells Showed Increased EMT and Decreased Apoptosis

**Figure 1:**
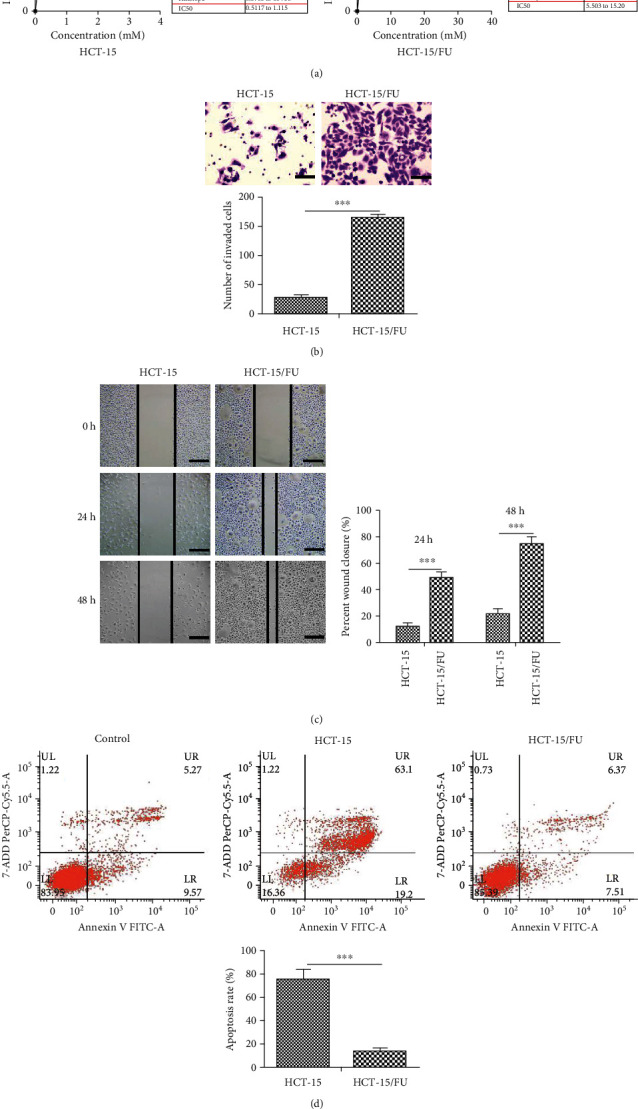
5-FU-resistant colon cancer cells showed increased EMT and decreased apoptosis. (a) MTS assay to detect inhibition of growth by 5-FU in HCT-15 or HCT-15/FU colon cancer cells. (b) and (c) The cell migration assay of HCT-15 or HCT-15/FU cells treated with 0.76 mM 5-FU. (b) Transwell migration assay (scale bars, 50 *μ*m) and (c) wound healing assay (scale bars, 200 *μ*m). (d) The flow cytometric apoptosis assays of HCT-15 or HCT-15/FU cells treated with 0.76 mM 5-FU. The data represent the mean ± SEM. Statistical significance: ^∗∗∗^*P* < 0.001.

We generated 5-FU-resistant HCT-15/FU cells from the 5-FU-sensitive parental HCT-15 cells by continuously exposing them to gradually increasing concentrations of 5-FU. We treated the 5-FU-sensitive and resistant cells with increasing doses of 5-FU for 48 h and examined the growth inhibition using MTS assays. As shown in Figure 1(a), the half maximal inhibitory concentration (IC_50_) value of 5-FU in HCT-15 and HCT-15/FU cells is 0.76 mM and 9.15 mM, respectively. We observed the migration and apoptosis of HCT-15 and HCT-15/FU cells after treating them with 0.76 mM 5-FU for 48 h. The 5-FU-resistant HCT-15/FU cells exhibited enhanced migration capacity as compared to the sensitive cells; this was confirmed by transwell and wound healing experiments (Figures 1(b) and 1(c)). In contrast, the apoptosis of HCT-15/FU cells was reduced after 5-FU treatment (Figure 1(d)). These results suggested that 5-FU-resistant colon cancer cells show enhanced migration and antiapoptotic ability.

### 3.2. The Low Expression of GDF15 in 5-FU-Resistant Cells Was Associated with Enhanced Migration and Antiapoptotic Ability

**Figure 2:**
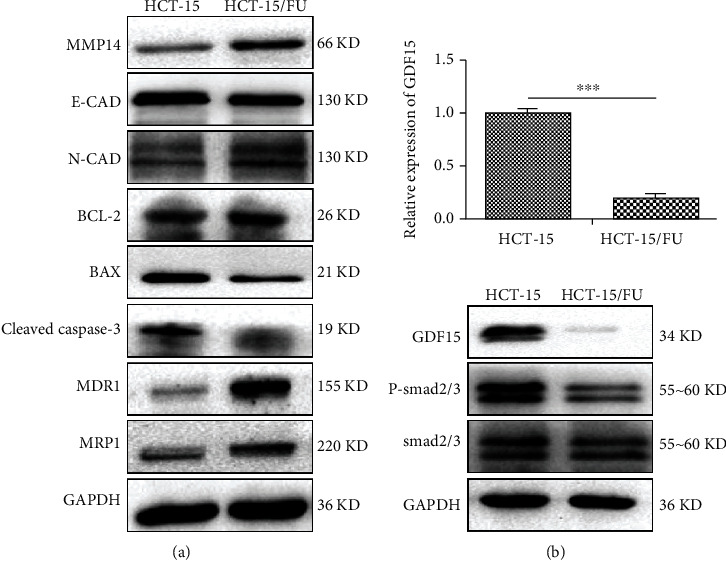
The low expression of GDF15 in 5-FU-resistant cells was associated with enhanced migration and antiapoptotic ability. (a) Western blotting analysis of MDR1, MRP1, MMP14, N-cadherin, E-cadherin, Bcl-2, cleaved caspase-3, and Bax levels in HCT-15 and HCT-15/FU cells treated with 0.76 mM 5-FU for 48 hours. (b) GDF15 mRNA and protein level in HCT-15 and HCT-15/FU cells treated with 0.76 mM 5-FU for 48 h. Upper: the relative mRNA expression of GDF15 in HCT-15 and HCT-15/FU cells; lower: western blotting analysis of GDF15 and the downstream signaling pathway protein P-Smad2/3 in HCT-15 and HCT-15/FU cells. The data represent the mean ± SEM. Statistical significance: ^∗∗∗^*P* < 0.001.

As shown in Figure 2(a), 5-FU-resistant colon cells expressed a high level of multidrug-resistant proteins (MDR1 and MRP1). The increased migration and antiapoptotic properties of HCT-15/FU cells were correlated with increased expression levels of N-cadherin, B-cell lymphoma 2 (Bcl-2), and matrix metallopeptidase 14 (MMP14) and decreased expression levels of the E-cadherin, cleaved caspase-3, and Bcl-2-associated X protein (BAX), as compared to HCT-15 cells (Figure 2(a)). These results further verified that 5-FU-resistant colon cancer cells had enhanced migration and antiapoptotic ability.

Our previous studies revealed that GDF15 was significantly higher in exosomes of HCT-15/FU cells as compared to exosomes of HCT-15 cells. We next investigated intracellular levels of GDF15. The mRNA and protein levels of GDF15 were decreased in the HCT-15/FU cells as compared with HCT-15 cells (Figure 2(b)). As a consequence of being a member of the BMP/TGF-*β* superfamily, as shown in Figure 2(b), the low expression of GDF15 in HCT-15/FU cells was accompanied by a reduction of P-Smad2/3. Therefore, we suspected that the inhibition of GDF15 and related Smad signaling pathways was associated with improved migration and antiapoptotic ability of 5-FU-resistant cells.

### 3.3. The Overexpression of GDF15 Resensitized the 5-FU-Resistant HCT-15/FU Cells to 5-FU

**Figure 3:**
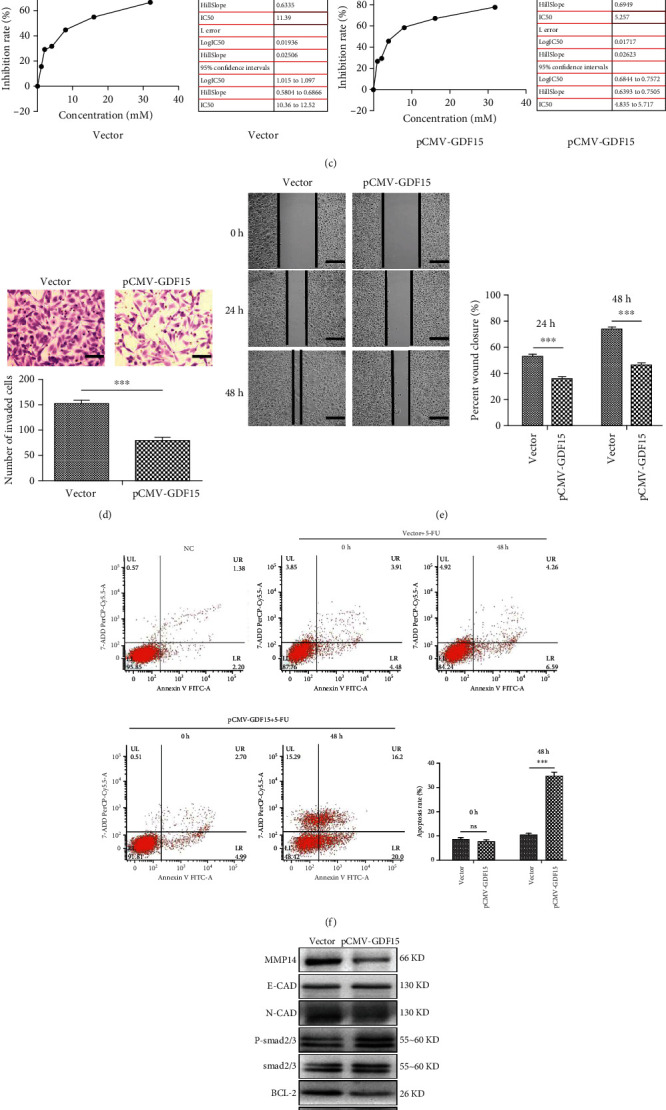
The overexpression of GDF15 resensitized the 5-FU-resistant HCT-15/FU cells to 5-FU. (a) The mRNA expression of GDF15 in HCT-15/FU cells treated with 0.76 mM 5-FU for 0 h or 48 h after being transfected with pCMV-GDF15 or empty vector. (b) Western blotting analysis of GDF15 in HCT-15/FU cells treated with 0.76 mM 5-FU for 0 h or 48 h after being transfected with pCMV-GDF15 or empty vector. (c) The MTS assay to detect inhibition of growth by 5-FU in HCT-15/FU cells treated with 5-FU after being transfected with pCMV-GDF15 or empty vector. (d, e) The cell migration assay of HCT-15/FU cells treated with 0.76 mM 5-FU for 0 h or 48 h after being transfected with pCMV-GDF15 or empty vector. (d) The transwell migration assay (scale bars, 50 *μ*m) and (e) wound healing assay (scale bars, 200 *μ*m). (f) The flow cytometric apoptosis assays of HCT-15/FU cells treated with 0.76 mM 5-FU for 0 h or 48 h after being transfected with pCMV-GDF15 or empty vector. (g) Western blotting analysis of MDR1, MRP1, MMP14, N-cadherin, E-cadherin, Bcl-2, cleaved caspase-3, and Bax levels in HCT-15/FU cells treated with 0.76 mM 5-FU for 48 h after being transfected with pCMV-GDF15 or empty vector. The data represent the mean ± SEM. Statistical significance: ^∗∗∗^*P* < 0.001.

To investigate the direct role of GDF15 in 5-FU resistance, we first restored the expression of GDF15 in 5-FU-resistant cells by transfecting them with a GDF15-overexpressing plasmid (pCMV-GDF15). The GDF15 expression remained high and stable in 5-FU-resistant cells, as compared to the empty vector transfection, even after the sustained action of 5-FU for 48 h (Figures 3(a) and 3(b)). As is shown in Figure 3(c), the upregulation of GDF15 could reverse the 5-FU resistance of HCT-15/FU cells: the IC_50_ of 5-FU was reduced from 11.39 mM to 5.26 mM. After treatment with 5-FU for 48 h, the migration and antiapoptotic ability of HCT-15/FU in the GDF15 overexpressed group were significantly hindered, compared to the control group (Figures 3(d)–3(f)). Western blotting revealed that the GDF15 upregulation reversed the elevated expression levels of MDR1, MRP1, N-cadherin, Bcl-2, and MMP14 and enhanced the expression levels of E-cadherin, BAX, cleaved caspase-3, and P-Smad2/3 in HCT-15/FU cells (Figure 3(g)). These results suggested that the low expression of GDF15 enhanced the migration and antiapoptosis ability of colon cancer cells and that the overexpression of GDF15 could partially reverse these effects by activating the Smad signaling pathway.

### 3.4. HCT-15/FU Cells Showed a Stronger Tendency to Proliferate and Metastasize In Vivo

**Figure 4:**
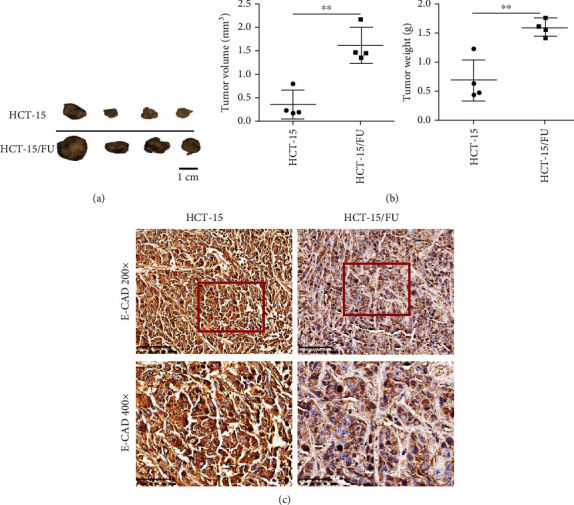
HCT-15/FU cells showed a stronger tendency to proliferate and metastasize in vivo. (a) The appearance of the subcutaneous tumors in mice treated with HCT-15 cells (1 × 10^7^) or HCT-15/FU cells (1 × 10^7^) treated with 0.76 mM 5-FU. (b) Weight and volume of the subcutaneous tumors (a). (c) The expression of E-cadherin in the subcutaneous tumors (a) was assessed by immunohistochemical staining. Statistical significance: ^∗∗∗^*P* < 0.001.

An in vivo model was created by injecting HCT-15 and HCT-15/FU cells into nude mice, along with injections of 5-FU (0.76 mM) every other day. The in vivo results were consistent with the in vitro results. The HCT-15/FU group had higher values of tumor volume and tumor weight and the lower E-cadherin expression as compared to the HCT-15 group (Figure 4). Therefore, in the presence of 5-FU treatment, the HCT-15/FU cells with low GDF15 expression exhibited a more robust proliferation and a greater capacity for metastasis.

## 4. Discussion

Resistance to 5-FU is a major obstacle in the treatment of colon cancer. Therefore, it is critical to understand the mechanisms of 5-FU resistance. Drug-resistant cells exhibit accelerated proliferation and distant metastasis [11–13]. Consistent with previous studies, the present findings demonstrate that 5-FU resistance in colon cancer cells is mechanistically associated with their enhanced migration and antiapoptotic ability by inhibiting the expression of GDF15 and related signaling pathways.

EMT is a dynamic process that is essential for the development of multicellular organisms. Its influence on tumor progression is clearly established [14]. Increasing evidence suggests that EMT can enable tumor cells to acquire metastatic features and develop therapeutic resistance and immune escape ability [15]. Our results indicated that HCT-15/FU cells underwent EMT (through the modulation of the expression of N-cadherin, E-cadherin, and MMP14), which is related to the inhibition of the Smad signaling pathway. In vivo experiments also confirmed the absence of the epithelial marker (E-cadherin) in the HCT-15/FU group. Whether drug-resistant colon cancer cells are more prone to distant metastasis, such as to the liver, lungs, and peritoneum, via EMT needs a further study.

5-FU plays an antitumor role via inhibition of thymine nucleotide synthase and then by interfering with DNA synthesis. Under the effect of chemotherapy drugs, tumor cells are subjected to stress damage and appear apoptotic [16]. In this study, we found that upon 5-FU treatment, the apoptosis of HCT-15/FU cells was significantly reduced, compared to HCT-15 cells; this was further confirmed by changes in apoptotic executive proteins (Bcl-2, BAX, and cleaved caspase-3). These results suggested that apoptosis is associated with 5-FU resistance in colon cancer cells. However, the regulatory network of apoptosis is complex and involves multiple processes and a cascade of reactions. Identifying the critical regulatory points of apoptosis involved in drug resistance will have profound significance in reversing drug resistance in colon cancer and enhancing treatment. Future studies should pay more attention to this aspect.

Evidence suggests that GDF15 plays different roles in different stages of tumor progression [17]. It inhibits early tumor promotion but accelerates tumor progression in advanced cancers [17]. It can also promote drug or radiation resistance in tumor cells by acting on the Smad signaling pathway [9]. Previous studies have confirmed that GDF15 is closely associated with tumor stem cells and can be used as a marker to reflect the prognosis of colon cancer [7, 18, 19]. In our study, we found that 5-FU resistance was caused due to the inhibition of GDF15 and the downstream Smad signaling pathway in colon cancer. The overexpression of GDF15 can resensitize 5-FU-resistant HCT-15/FU cells to 5-FU. Our in vivo experiment also validated that HCT-15/FU with low GDF15 expression had more robust proliferation capacity and metastasis tendency. These results demonstrate that GDF15 might act as a tumor suppressor gene in colon cancer. Many mechanisms result in the inactivation of tumor suppressor genes, such as loss of heterozygosity [20–22], DNA methylation [23, 24], or acetylation [25, 26]. Future studies should focus on the mechanism through which GDF15 is repressed in these cells; this finding could provide new strategies for the diagnosis and gene therapy of colon cancer.

## 5. Conclusion

Overall, our findings suggest that 5-FU resistance in colon cancer cells is associated with the repression of GDF15 and the subsequent enhancement of the EMT and antiapoptotic ability of cancer cells. Although we did not show the mechanism of GDF15 repression, we have provided evidence for its significance in 5-FU resistance in colon cancer cells and identified a gene therapy target for reversing drug resistance and improving the prognosis of colon cancer patients.

## Data Availability

All data generated or analyzed during our study are available from the corresponding author on reasonable request.

## Abbreviations

5-FU: 5-Fluorouracil
GDF15: Growth differentiation factor 15
EMT: Epithelial-mesenchymal transition
MDR1: Multiple drug resistance
MRP1: Multidrug resistance-related protein
N-CAD: N-Cadherin
E-CAD: E-Cadherin
Bcl-2: B-cell lymphoma 2
MMP14: Matrix metallopeptidase 14
BAX: Bcl-2-associated X protein
